# Supplementary material for: How facemasks shape trust in social interactions
Source: PLoS One. 2025 Sep 12;20(9):e0331918. doi: 10.1371/journal.pone.0331918 (PMC12431196; doi:10.1371/journal.pone.0331918)

**S1 Face stimuli selection**

The images were taken from the Chicago Face Database. We first selected all faces showing a neutral expression. Within these neutral images, for each image labelled as ‘white male’, we computed the normalised age, attractiveness rating and trustworthiness rating. We did the same for the images labelled ‘white female’. We then randomly selected four male and four female pictures from the set of images for which the squared deviation from the median for any given measure did not exceed 0.001, and the sum of the squared deviations did not exceed 0.002. The reference number of all faces used in the paper are WF-001, -006, -020, and -038 for white females, and WM-038, -040, -218, and -229 for white males (Pictures below are presented in sequence).


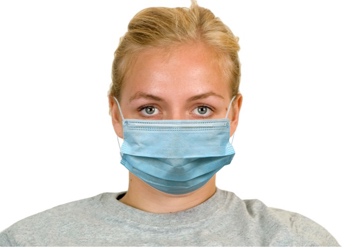

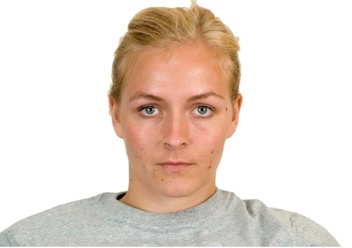


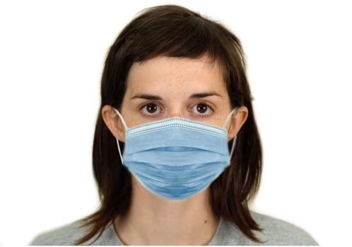

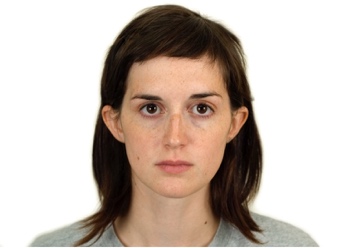


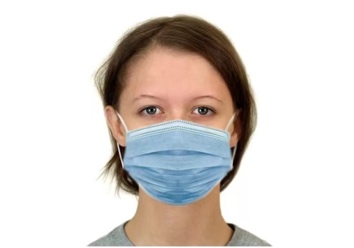

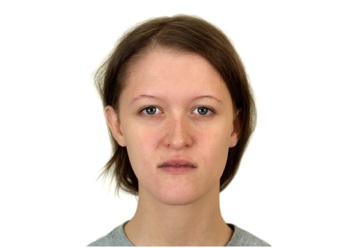


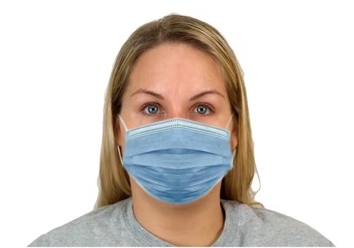

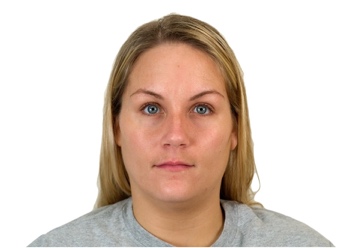


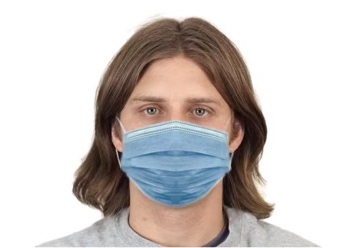

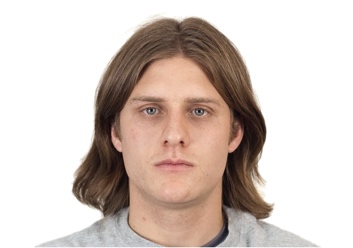


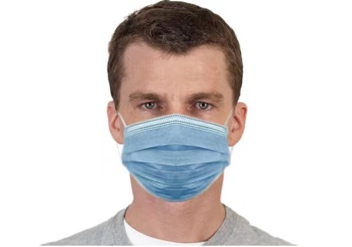

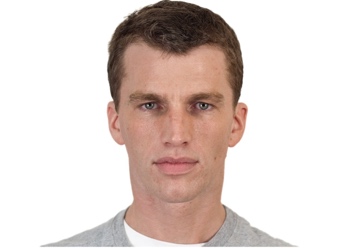


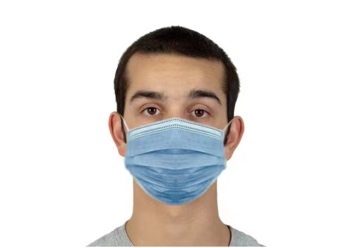

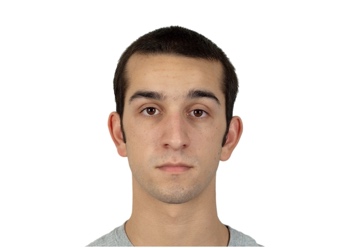


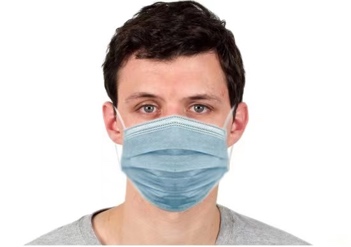

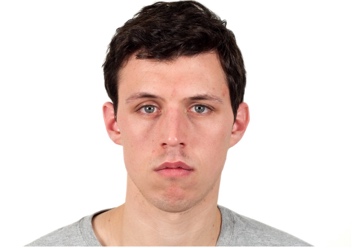

Supplement: S1 File — (DOCX) [file pone.0331918.s001.docx]
