# Supplementary material for: How facemasks shape trust in social interactions
Source: PLoS One. 2025 Sep 12;20(9):e0331918. doi: 10.1371/journal.pone.0331918 (PMC12431196; doi:10.1371/journal.pone.0331918)
Supplement: S2 File — (DOCX) [file pone.0331918.s002.docx]

**S2 Regression tables of exploratory analysis**

**Table S2.1 Linear regression predicting the amount sent by trustors in Experiments 1 and 2, with additional/exploratory predictors.**

|  | Experiment 1 | | | Experiment 2 | | |
| --- | --- | --- | --- | --- | --- | --- |
| Predictors | Estimates | CI | p | Estimates | CI | p |
| Masked picture | 0.69  (0.55) | [–0.70, 2.19] | 0.311 | –1.34  (1.04) | [–3.39, 0.70] | 0.198 |
| Male participants | 1.41  (0.46) | [0.49, 2.30] | 0.002 | 0.54  (0.56) | [–0.57, 1.65] | 0.336 |
| Male picture | 0.19  (0.45) | [–0.66, 1.12] | 0.617 | - | - | - |
| Played trustor first | 0.57  (0.45) | [–0.24, 1.53] | 0.153 | 1.30  (0.59) | [0.14, 2.46] | 0.028 |
| Trustworthiness Rating | 0.04  (0.01) | [0.03, 0.06] | <0.001 | 0.03  (0.01) | [0.00, 0.05] | 0.019 |
| Facemask protection attitude: |  |  |  |  |  |  |
| Only/mostly others | 0.60  (0.32) | [–0.04, 1.23] | 0.065 | –0.09  (0.52) | [–1.11, 0.93] | 0.864 |
| Only/mostly themselves | –0.53  (0.26) | [–1.04, –0.02] | 0.043 | –0.013  (0.37) | [–0.86, 0.61] | 0.732 |
| Facemask wearing behaviour: |  |  |  |  |  |  |
| Less frequent | - | - | - | –0.68  (0.34) | [–1.35, –0.02] | 0.044 |
| Male picture * Masked picture | –0.29  (0.45) | [–1.21, 0.57] | 0.482 | - | - | - |
| Male participants * Masked picture | –0.45  (0.45) | [–1.29, 0.49] | 0.377 | 0.27  (0.67) | [–1.04, 1.58] | 0.686 |
| Male participants * Male picture | –0.25  (0.45) | [–1.13, 0.64] | 0.587 | - | - | - |
| Masked picture * Played trustor first | 0.30  (0.45) | [–0.65, 1.13] | 0.592 | 0.67  (0.66) | [–0.64, 1.98] | 0.314 |
| Male picture * Played trustor first | 0.02  (0.45) | [–0.86, 0.91] | 0.961 | - | - | - |
| Male participants * Played trustor first | 0.51  (0.45) | [–0.40, 1.37] | 0.285 | –0.00  (0.67) | [–1.31, 1.31] | 0.996 |
| Masked picture * Trustworthiness | –0.02  (0.01) | [–0.04, 0.01] | 0.217 | 0.02  (0.02) | [–0.02, 0.05] | 0.328 |
| Observations | 718 | | | 361 | | |
| R^2^ | 0.137 | | | 0.124 | | |
| Adj. R^2^ | 0.120 | | | 0.096 | | |
| AIC | 3633.0 | | | 1859.8 | | |

**Table S2.2 Multi-level regression predicting the proportion returned by trustees in Experiment 1 and 2, with additional/exploratory predictors.**

|  | Experiment 1 | | | Experiment 2 | | | |
| --- | --- | --- | --- | --- | --- | --- | --- |
| Predictors | Estimates | CI | p | Estimates | | CI | p |
| Masked picture | 0.003  (0.02) | [–0.04, 0.04] | 0.880 | –0.01  (0.02) | [–0.05, 0.04] | | 0.805 |
| Male participant | 0.01  (0.02) | [–0.03, 0.06] | 0.494 | –0.004  (0.02) | [–0.05, 0.04] | | 0.851 |
| Male picture | –0.001  (0.02) | [–0.04, 0.04] | 0.962 | - | - | | - |
| Played trustor first | -0.03  (0.01) | [–0.05, –0.00] | 0.026 | 0.001  (0.02) | [–0.03, 0.04] | | 0.942 |
| Amount Sent | 0.01  (0.002) | [0.01, 0.02] | <0.001 | 0.01  (0.003) | [0.00, 0.01] | | 0.019 |
| Trustworthiness Rating | 0.002  (0.001) | [0.001, 0.003] | <0.001 | 0.002  (0.001) | [0.001, 0.002] | | <0.001 |
| Facemask protection attitude: |  |  |  |  |  | |  |
| Only/mostly other | 0.01  (0.02) | [–0.02, 0.05] | 0.486 | 0.02  (0.03) | [–0.03, 0.07] | | 0.446 |
| Only/mostly themselves | 0.06  (0.01) | [–0.02, 0.03] | 0.662 | –0.01  (0.02) | [–0.04, 0.03] | | 0.728 |
| Facemask wearing behaviour: |  |  |  |  |  | |  |
| Less frequent | - | - | - | –0.03  (0.02) | [–0.06, 0.01] | | 0.099 |
| Male picture * Masked picture | –0.01  (0.02) | [–0.06, 0.04] | 0.637 | - | - | | - |
| Male participant * Masked picture | –0.01  (0.02) | [–0.06, 0.03] | 0.547 | –0.008  (0.03) | [–0.08, 0.06] | | 0.799 |
| Male participant * Male picture | 0.004  (0.02) | [–0.04, 0.05] | 0.883 | - | - | | - |
| Observations | 7180 | | | 3610 | | | |
| Marginal R^2^ | 0.105 | | | 0.068 | | | |
| Conditional R^2^ | 0.770 | | | 0.722 | | | |
| AIC | –10628.7 | | | –4610.5 | | | |

**Table S2.3 Linear regression predicting perceived trustworthiness in Experiments 1 and 2 as a function of participant’s gender (Male participant), counterpart’s mask status (Masked picture), counterpart’s gender (Male picture), the order in which the participant completed the tasks (Played trustor first), and attitudes to facemasks.**

|  | Experiment 1 | | | Experiment 2 | | | |
| --- | --- | --- | --- | --- | --- | --- | --- |
| Predictors | Estimates | CI | p | Estimates | CI | p | |
| Masked picture | 8.32  (2.75) | [2.93, 13.71] | 0.003 | 9.95  (3.61) | [2.85, 17.05] | 0.006 |  |
| Male participant | -3.14  (2.74) | [–8.52, 2.25] | 0.253 | 0.91  (3.653) | [–6.29, 8.08] | 0.806 |  |
| Male picture | -7.63  (2.74) | [–13.02, –2.24] | 0.006 | - | - | - |  |
| Played trustor first | 1.39  (2.71) | [–3.93, 6.71] | 0.607 | -3.32  (3.578) | [–10.36, 3.72] | 0.355 |  |
| Facemask protection attitude: |  |  |  |  |  |  |  |
| Mostly other | 1.85  (1.97) | [–2.01, 5.71] | 0.346 | 1.27  (3.287) | [–5.19, 7.74] | 0.699 |  |
| Mostly themselves | -2.78  (1.61) | [–5.95, 0.38] | 0.085 | -1.24  (2.398) | [–5.96, 3.47] | 0.605 |  |
| Only others | -0.141  (8.18) | [–16.20, 15.92] | 0.986 | 24.41  (14.119) | [–3.36, 52.18] | 0.085 |  |
| Only themselves | -6.466  (4.05) | [–14.42, 1.49] | 0.111 | -4.73  (5.712) | [–15.97, 6.50] | 0.408 |  |
| Facemask wearing behaviour: |  |  |  |  |  |  |  |
| Much less frequently | - | - | - | -4.33  (2.798) | [–9.83, 1.18] | 0.123 |  |
| Less frequently | - | - | - | -1.28  (2.505) | [–6.21, 3.65] | 0.609 |  |
| Much more frequently | - | - | - | 0.56  (5.376) | [–10.02, 11.13] | 0.917 |  |
| More frequently | - | - | - | -6.87  (5.415) | [–17.52, 3.79] | 0.206 |  |
| Male picture * Masked picture | 5.18  (2.70) | [–0.13, 10.48] | 0.056 | - | - | - |  |
| Male participant * Masked picture | 1.61  (2.70) | [–3.70, 6.92] | 0.552 | - | - | - |  |
| Male participant * Male picture | 5.19  (2.70) | [–0.11, 10.49] | 0.055 | - | - | - |  |
| Masked picture* Played trustor first | 1.33  (2.71) | [–3.98, 6.65] | 0.622 | 7.11  (4.121) | [–1.00, 15.21] | 0.086 |  |
| Male picture * Played trustor first | -1.66  (2.70) | [–6.97, 3.65] | 0.540 | - | - | - |  |
| Male participant * Played trustor first | 1.90  (2.70) | [–3.42, 7.22] | 0.483 | 0.82  (4.153) | [–7.35, 8.99] | 0.844 |  |
| Observations | 718 | | | 361 | | |  |
| R^2^ | 0.050 | | | 0.054 | | |  |
| Adj. R^2^ | 0.031 | | | 0.016 | | |  |
| AIC | 6208.2 | | | 3181.4 | | |  |
