# Supplementary material for: How facemasks shape trust in social interactions
Source: PLoS One. 2025 Sep 12;20(9):e0331918. doi: 10.1371/journal.pone.0331918 (PMC12431196; doi:10.1371/journal.pone.0331918)
Supplement: S3 File — (DOCX) [file pone.0331918.s003.docx]

**S3 Sample size using different exclusion criteria and comprehension questions**

**Table S3.1 Sample size using different exclusion criteria**

|  | Exp 1 | Exp 2 |
| --- | --- | --- |
| Apply the first two comprehension questions: | 718/804 response left | 361/402 response left |
| Apply the two advanced comprehension questions: | 464/804 response left | 243/402 response left |
| Apply all four comprehension questions: | 451/804 response left | 235/402 response left |
| Notice: Across three exclusion criteria, we excluded participants who answered any of the questions incorrectly. | | |

**Comprehension questions for Exp 1 & 2**

1. Imagine the Sender sends £3.

How much does this become before it is received by the Responder?

1. £3
2. £9🗸
3. £8
4. £13
5. Now suppose that, after receiving the money from the Sender, the Responder returns £2.

How much does the Sender receive?

1. £2🗸
2. £6
3. £12
4. £15

**Advanced comprehension questions for Exp 1 & 2**

1. Based on the information from the previous questions, and recalling that the Sender received £10 at the beginning of the interaction, how much do they earn in total?
   1. £9 (Initial £10 minus £3 sent plus £2 received from Responder) 🗸
   2. £13 (Initial £10 minus £3 sent plus £6 received from Responder)
   3. £19 (Initial £10 minus £3 sent plus £12 received from Responder)
   4. £22 (Initial £10 minus £3 sent plus £15 received from Responder)
2. Based on the information from the previous questions, and recalling that the Responder received £10 at the beginning of the interaction, how much do they earn in total?
   1. £17 (Initial £10 plus £9 received from Sender minus £2 returned) 🗸
   2. £11 (Initial £10 plus £3 received from Sender minus £2 returned)
   3. £16 (Initial £10 plus £8 received from Sender minus £2 returned)
   4. £21 (Initial £10 plus £13 received from Sender minus £2 returned)

**Comprehension question for Exp 3**

How much would the person you selected on the previous page receive?

1. £20
2. £10
3. £15
4. £30
