# Supplementary material for: How facemasks shape trust in social interactions
Source: PLoS One. 2025 Sep 12;20(9):e0331918. doi: 10.1371/journal.pone.0331918 (PMC12431196; doi:10.1371/journal.pone.0331918)
Supplement: S4 File — (DOCX) [file pone.0331918.s004.docx]

**S4 Regression results with sample size after applying all four comprehension questions**

**Table S4.1 Linear regression on the amount sent by trustors, using stricter exclusion criteria***.*

|  | Experiment 1 | | | Experiment 2 | | |
| --- | --- | --- | --- | --- | --- | --- |
| Predictors | Estimates | CI | p | Estimates | CI | p |
| Masked picture | 0.46  (0.49) | [-0.51, 1.43] | 0.356 | 0.04  (0.58) | [–1.09, 1.18] | 0.940 |
| Male participant | 1.40  (0.49) | [0.42, 2.37] | 0.005 | 0.48  (0.57) | [–0.46, 1.80] | 0.244 |
| Male picture | 0.06  (0.49) | [–0.89, 1.02] | 0.899 | - | - | - |
| Played trustor first | 0.93  (0.29) | [0.37, 1.50] | 0.001 | 1.15  (0.41) | [0.35, 1.95] | 0.005 |
| Male picture * Masked picture | -0.74  (0.57) | [–1.87, 0.38] | 0.195 | - | - | - |
| Male participant * Masked picture | 0.34  (0.57) | [–0.79, 1.47] | 0.554 | 0.19  (0.81) | [–1.79, 1.41] | 0.814 |
| Male participant * Male picture | -0.26  (0.57) | [–1.39, 0.86] | 0.646 | - | - | - |
| Observations | 451 | | | 235 | | |
| R^2^ | 0.083 | | | 0.042 | | |
| Adj. R^2^ | 0.069 | | | 0.025 | | |
| AIC | 2293.4 | | | 1202.2 | | |

**Table S4.2 Multi-level regression on the proportion returned by trustees using stricter exclusion criteria.**

|  | Experiment 1 | | | Experiment 2 | | |
| --- | --- | --- | --- | --- | --- | --- |
| Predictors | Estimates | CI | p | Estimates | CI | p |
| Masked picture | –0.01  (0.024) | [–0.06, 0.04] | 0.694 | –0.02  (0.027) | [–0.08,0.03] | 0.406 |
| Male participant | 0.01  （0.022） | [–0.05, 0.05] | 0.936 | –0.02  (0.029) | [–0.08, 0.03] | 0.412 |
| Male picture | –0.03  (0.024) | [–0.07, 0.02] | 0.255 | - | - | - |
| Played trustor first | –0.05  (0.014) | [–0.05, -0.02] | <0.001 | –0301  (0.020) | [–0.04, 0.05] | 0.680 |
| Amount Sent | 0.01  (0.003) | [0.01, 0.02] | <0.001 | 0.01  (0.004) | [0.00, 0.01] | 0.156 |
| Male picture * Masked picture | 0.01  (0.029) | [–0.04, 0.07] | 0.602 | - | - | - |
| Male participant * Masked picture | –0.02  (0.029) | [–0.07, 0.04] | 0.555 | 0.04  (0.040) | [–0.03, 0.12] | 0.268 |
| Male participant * Male picture | 0.02  (0.029) | [–0.03, 0.08] | 0.390 | - | - | - |
| Observations | 4510 | | | 2350 | | |
| Marginal R^2^ | 0.056 | | | 0.013 | | |
| Conditional R^2^ | 0.751 | | | 0.734 | | |
| AIC | –7121.5 | | | –3475.8 | | |
