# Supplementary material for: How facemasks shape trust in social interactions
Source: PLoS One. 2025 Sep 12;20(9):e0331918. doi: 10.1371/journal.pone.0331918 (PMC12431196; doi:10.1371/journal.pone.0331918)
Supplement: S5 File — (DOCX) [file pone.0331918.s005.docx]

**S5 Analysis of attractiveness ratings**

**Table S5.1 Linear regression predicting perceived attractiveness in Experiments 1 and as a function of participant’s gender (Male participant), counterpart’s mask status (Masked picture), and counterpart’s gender (Male picture).**

|  | Experiment 1 | | | Experiment 2 | | |
| --- | --- | --- | --- | --- | --- | --- |
| Predictors | Estimates | CI | p | Estimates | CI | p |
| Masked picture | 6.74  (2.70) | [1.44, 12.03] | 0.013 | 0.28  (3.01) | [–6.21, 5.65] | 0.926 |
| Male participant | –0.91  (2.70) | [6.21, 4.39] | 0.735 | -4.57  (3.01) | [–10.49, 1.35] | 0.129 |
| Male picture | –9.33  (2.67) | [–14.57, –4.08] | <0.001 | - | - | - |
| Male picture * Masked picture | -6.15  (3.13) | [–12.03, –0.00] | 0.049 | - | - | - |
| Male participant * Masked picture | –1.73  (3.13) | [–7.88, 4.42] | 0.581 | 1.40  (4.28) | [–9.81, 7.01] | 0.743 |
| Male participant * Male picture | 5.90  (3.13) | [-0.25, 12.05] | 0.060 | - | - | - |
| Observations | 718 | | | 361 | | |
| R^2^ | 0.063 | | | 0.017 | | |
| Adj. R^2^ | 0.055 | | | 0.009 | | |
| AIC | 6416.2 | | | 3202.7 | | |

In Exp 3, a 2 (masked vs unmasked pictures) by 2 (male vs female participants) mixed ANOVA was conducted to examine the effects of masks on attractiveness ratings. According to the results, there was neither a significant main effect of the facemask manipulation (F(169) = 0.99, p =.321), nor a significant interaction effect between facemask manipulation and participant’s gender (F(169) = 0.72, p = 0.399), which suggests that participants’ attractiveness ratings were not affected by whether the picture was presented with or without a facemask.

We observed moderate positive correlations between trustworthiness and attractiveness ratings across all experiments: Experiment 1 (r = 0.46), Experiment 2 (r = 0.39), and Experiment 3 (r = 0.53), indicating that higher attractiveness was generally associated with higher perceived trustworthiness.
