# Supplementary material for: How facemasks shape trust in social interactions
Source: PLoS One. 2025 Sep 12;20(9):e0331918. doi: 10.1371/journal.pone.0331918 (PMC12431196; doi:10.1371/journal.pone.0331918)
Supplement: S6 File — (DOCX) [file pone.0331918.s006.docx]

**S6** **Logistic regression results after re-parameterizing the dependent variable**

**Table S6.1 Logistic regression predicting whether the left counterpart received £20 based on Mask appearance, Gender, Mask wearing by participant, and difference in Trustworthiness rating between masked and unmasked counterpart.**

|  | Model 1 | | | Model 2 | | | Model 3 | | |
| --- | --- | --- | --- | --- | --- | --- | --- | --- | --- |
| Predictors | Odds Ratio | CI | p | Odds Ratios | CI | p | Odds Ratio | CI | p |
| (Intercept) | 0.84 | [0.60, 1.18] | 0.319 | 0.93 | [0.60, 1.43] | **0.936** | 0.92 | [0.50, 1.69] | 0.791 |
| Masked | 2.54 | [1.83, 3.60] | <0.001 | 1.84 | [1.24 2.74] | **0.002** | 2.99 | [1.74, 5.78] | **<0.001** |
| Gender | 0.80 | [0.57, 1.12] | 0.204 | 0.85 | [0.58, 1.25] | 0.412 | 0.79 | [0.52, 1.19] | 0.267 |
| Allocation scenario | 0.85 | [0.60, 1.19] | 0.338 | 0.92 | [0.62, 1.35] | 0.657 | 0.89 | [0.59, 1.33] | 0.563 |
| Mask wearing habit |  |  |  | 1.23 | [0.80, 1.90] | 0.343 | 1.19 | [0.65, 2.16] | 0.559 |
| Trustworthiness Difference |  |  |  | 1.11 | [1.06, 1.18] | **<0.001** | 1.11 | [1.06, 1.17] | **<0.001** |
| Masked * Gender |  |  |  |  |  |  | 1.41 | [0.94, 2.17] | 0.101 |
| Masked * Allocation scenario |  |  |  |  |  |  | 1.25 | [0.83, 1.89] | 0.289 |
| Masked * Mask wearing habit |  |  |  |  |  |  | 2.17 | [1.28, 4.14] | **0.008** |
| Observations | 171 | | | 171 | | | 171 | | |
| $R^{2}$ Tjur* | 0.197 | | | 0.384 | | | 0.447 | | |
| AIC | 209.264 | | | 171.332 | | | 164.815 | | |

* Tjur’s (2009) coefficient of determination.
