# Supplementary material for: How facemasks shape trust in social interactions
Source: PLoS One. 2025 Sep 12;20(9):e0331918. doi: 10.1371/journal.pone.0331918 (PMC12431196; doi:10.1371/journal.pone.0331918)
Supplement: S7 File — (DOCX) [file pone.0331918.s007.docx]

**S7 Linear probability regression results**

**Table S7.1 Linear probability regressions predicting whether the masked counterpart received £20 based on Gender, Mask wearing by participant, and difference in Trustworthiness rating between masked and unmasked counterpart.**

|  | Model 1 | | | Model 2 | | | Model 3 | | |
| --- | --- | --- | --- | --- | --- | --- | --- | --- | --- |
| Predictors | Estimates | CI | p | Estimates | CI | p | Estimates | CI | p |
| (Intercept) | 0.74 | [0.73, 1.02] | <0.001 | 0.88 | [0.73, 1.02] | <0.001 | 0.83 | [0.74, 0.92] | <0.001 |
| Gender | –0.03 | [–0.17, 0.11] | 0.668 | –0.08 | [–0.20, 0.04] | 0.204 | 0.01 | [–0.05, 0.08] | 0.652 |
| Frame | –0.03 | [–0.16, 0.11] | 0.706 | –0.06 | [–0.18, 0.06] | 0.330 | 0.03 | [–0.03, 0.10] | 0.340 |
| Mask wearing habit |  |  |  | –0.22 | [–0.36, –0.09] | **0.001** | 0.13 | [0.05, 0.20] | 0.001 |
| Trustworthiness Difference |  |  |  | 0.01 | [0.01, 0.01] | **<0.001** |  |  |  |
| Facemask protection attitude: |  |  |  |  |  |  |  |  |  |
| Only & Mostly others |  |  |  |  |  |  | 0.10 | [–0.13, 0.34] | 0.400 |
| Only & Mostly themselves |  |  |  |  |  |  | –0.20 | [–0.34, –0.06] | 0.006 |
| Observations | 171 | | | 171 | | | 171 | | |
| $R^{2}$ adjusted | –0.010 | | | 0.215 | | | 0.126 | | |
| AIC | 221.460 | | | 180.356 | | | 199.667 | | |
