# Supplementary material for: How facemasks shape trust in social interactions
Source: PLoS One. 2025 Sep 12;20(9):e0331918. doi: 10.1371/journal.pone.0331918 (PMC12431196; doi:10.1371/journal.pone.0331918)
Supplement: S8 File — (DOCX) [file pone.0331918.s008.docx]

**S8 Regression results restricted to participants who played the trustor first and participants who played the trustee first**

**Table S8.1 Linear regression on the amount sent by trustors, restricted to participants who played the trustor first**

|  | Experiment 1 | | | Experiment 2 | | |
| --- | --- | --- | --- | --- | --- | --- |
| Predictors | Estimates | CI | p | Estimates | CI | p |
| Masked picture | 0.37 | [–0.72, 1.46] | 0.509 | 0.48 | [–0.85, 1.82] | 0.477 |
| Male participant | 1.35 | [0.25, 2.46] | 0.016 | 0.88 | [–0.51, 2.26] | 0.213 |
| Male picture | 0.23 | [–0.88, 1.34] | 0.682 | - | - | - |
| Male picture * Masked picture | –1.04 | [–2.34, 0.26] | 0.118 | - | - | - |
| Male participant * Masked picture | 0.41 | [–0.90, 1.71] | 0.541 | –0.21 | [–2.14, 1.72] | 0.830 |
| Male participant * Male picture | 0.14 | [–1.15, 1.46] | 0.814 | - | - | - |
| Observations | 366 | | | 178 | | |
| $\boldsymbol{R}^{\boldsymbol{2}}$ | 0.071 | | | 0.017 | | |
| $\boldsymbol{Adj. R}^{\boldsymbol{2}}$ | 0.055 | | | 0.004 | | |
| AIC | 1890.3 | | | 929.7 | | |

**Table S8.2 Multi-level regression on the proportion returned by trustees, restricted to participants who played the trustee first**

|  | Experiment 1 | | | Experiment 2 | | |
| --- | --- | --- | --- | --- | --- | --- |
| Predictors | Estimates | CI | p | Estimates | CI | p |
| Masked picture | –.004 | [–0.06, 0.05] | 0.907 | .019 | [–0.06,0.09] | 0.613 |
| Male participant | –.003 | [–0.08, 0.04] | 0.421 | .003 | [–0.07, 0.8] | 0.925 |
| Male picture | –.024 | [–0.06, 0.06] | 0.992 | - | - | - |
| Amount sent | .015 | [0.01, 0.02] | <.001 | .012 | [.002, 0.02] | 0.014 |
| Male picture * Masked picture | .002 | [–0.07, 0.02] | 0.997 | - | - | - |
| Male participant * Masked picture | –.001 | [–0.08, 0.06] | 0.829 | –.031 | [–0.13, 0.07] | 0.546 |
| Male participant * Male picture | .038 | [–0.03, 0.11] | 0.283 | - | - | - |
| Observations | 3520 | | | 1830 | | |
| Marginal $\boldsymbol{R}^{\boldsymbol{2}}$ | 0.056 | | | 0.033 | | |
| Conditional $\boldsymbol{R}^{\boldsymbol{2}}$ | 0.803 | | | 0.784 | | |
| AIC | –5613.7 | | | –2697.1 | | |
