# Supplementary material for: How facemasks shape trust in social interactions
Source: PLoS One. 2025 Sep 12;20(9):e0331918. doi: 10.1371/journal.pone.0331918 (PMC12431196; doi:10.1371/journal.pone.0331918)
Supplement: S9 File — (DOCX) [file pone.0331918.s009.docx]

**S9 Analysis at each transfer level**

To assess whether the facemask manipulation had any effect at specific transfer levels, we re-analysed the data from Experiments 1 and 2 by conducting separate linear regressions at each transfer level measured in the strategy method. Specifically, for each level of the amount sent by the trustor (i.e., £0 to £10, tripled to £0 to £30), we examined whether the proportion of money trustees indicated they would return was significantly predicted by the facemask condition (along with gender of the counterpart and role order as covariates). Across all levels, we found no statistically significant effect of the facemask condition on the proportion returned (all ps > .05), consistent with our main analysis. We have included the full regression output table in the following (Table S12) for transparency.

**Table S9.1 Regressions on the proportion returned by trustees on each transfer level**

|  | Experiment 1 | Experiment 2 |
| --- | --- | --- |
| Trustee response when receiving £3 | Estimates | Estimates |
| Predictor (Masked picture) | 0.03  [–0.09, 0.14] | –0.04  [–0.21, 0.13] |
| Trustee response when receiving £6 | Estimates | Estimates |
| Predictor (Masked picture) | 0.07  [–0.12, 0.26] | –0.11  [–0.38, 0.17] |
| Trustee response when receiving £9 | Estimates | Estimates |
| Predictor (Masked picture) | 0.04  [–0.23, 0.31] | –0.04  [–0.43, 0.35] |
| Trustee response when receiving £12 | Estimates | Estimates |
| Predictor (Masked picture) | –0.01  [–0.35, 0.33] | –0.03  [–0.52, 0.47] |
| Trustee response when receiving £15 | Estimates | Estimates |
| Predictor (Masked picture) | 0.04  [–0.37, 0.45] | 0.04  [–0.53, 0.60] |
| Trustee response when receiving £18 | Estimates | Estimates |
| Predictor (Masked picture) | 0.01  [–0.48, 0.49] | –0.08  [–0.76, 0.59] |
| Trustee response when receiving £21 | Estimates | Estimates |
| Predictor (Masked picture) | –0.01  [–0.58, 0.56] | 0.05  [–0.76, 0.86] |
| Trustee response when receiving £24 | Estimates | Estimates |
| Predictor (Masked picture) | –0.08  [–0.71, 0.56] | –0.25  [–1.19, 0.69] |
| Trustee response when receiving £27 | Estimates | Estimates |
| Predictor (Masked picture) | –0.20  [–0.96,0.55] | 0.07  [–1.02, 1.15] |
| Trustee response when receiving £30 | Estimates | Estimates |
| Predictor (Masked picture) | –0.13  [–0.92, 0.66] | 0.01  [–1.21, 1.23] |

with 95% CI between parentheses
