# Supplementary material for: How facemasks shape trust in social interactions
Source: PLoS One. 2025 Sep 12;20(9):e0331918. doi: 10.1371/journal.pone.0331918 (PMC12431196; doi:10.1371/journal.pone.0331918)
Supplement: Attachment — Submitted filename: D25-8765-Reviewer.pdf [file pone.0331918.s010.pdf]

How Facemasks Shape Trust in Social Interactions – Reviewer Report

Summary

The authors present three experiments using trust games (and a final variation with two simultaneous recipients) and varying whether the avatar with whom they played wore a facemask or not, and the sex of both roles in the game. Participants made choices in the role of the first (the trustee) and second mover (the trustor). In Experiments 1 and 2, using a standard trust game, the authors found no statistically significant effect of the facemask on the participants' hypothetical decisions in the game, although avatars wearing a mask were rated as more trustworthy. The variation in Experiment 3, with two simultaneous second movers —one with a facemask and one without —allowed the researchers to observe that the masked individual received the largest transfer more often.

Overview

I believe this manuscript addresses an important question (i.e., how wearing masks affects trust and trustworthiness in social interactions) and adds multiple evidence (though mixed) throughout three studies. I liked the alternative version of the trust game in which the first mover chooses how much to transfer to the two different second movers, but why it remains a trust game is poorly explained. I also have some concerns about hypothetical decisions and how artificial the stimuli may have looked. Nonetheless, I think the paper is worth being published in PLOS ONE after some adjustments that I propose below.

Main comments

1. **Concerns on the use of faces from a databank and hypothetical games.**

My main concern with the overall manuscript is an alternative explanation for why Experiments 1 and 2 yielded null results: the combination of hypothetical choices with pre-existing faces where the facemask was superimposed may have created an artificial decision environment, and respondents did not take it seriously.

1.1. Did you ask prolific respondents if they were familiar with these faces? As they are well-trained participants for this type of study, if they perceive the task as being too artificial, you may have more noisy responses that ultimately decrease your ability to detect effects. Please clarify if this question was asked, and discuss this in the paper.

1.2. I also believe that this issue of how artificial the decision looked may partly explain why those who decided first as the trustors were less prosocial: starting with the second decision reinforced the hypothetical nature of this trust game, and therefore, they could feel that their choices were less consequential toward others.

1.3. The use of the strategy method in the trust game could also explain the behavior of the second mover, reinforcing why those starting with this choice behaved differently. See Johnsen and Kvaløy (2016).

1.4. I also recommend that the authors justify their choice of a hypothetical trust game based on existing literature following the same incentive decision.

2. Design choices to explain better.

2.1. The design in Experiment 3 is interesting, but it must be better explained. Although the use of two simultaneous second movers is clever, the fact that the transferred amount does not increase must be better explained. At first sight, this game variation does not measure trust but instead generosity because there is no reason to give money back if it is purely redistributive. The key issue with the game is that the sender has no money left, so she *trusts* that the recipient of the largest amount would send some money back. This must be made clearer in the text.

2.2. In Experiments 1 and 2, it is not clear why participants played the trust game a single time. This limited the authors' possibilities of computing some helpful within-subjects measurements. Learning how to play the trust game was a high cost for respondents, so that they could then apply it more than once. I would like to hear the authors' thoughts on this.

2.3. Why—in Experiment 2—the sex restriction kept female counterparts instead of male counterparts? Please explain.

2.4. In Experiment 2, the 10-second exposure raises salience of the mask, but it may have also raised the experimenter demand effect. A discussion on this would help the reader clarify this design feature.

3. Statistical analysis

3.1. Is the effect of the facemask null for every transfer level? I suggest reporting, in the appendix or at least in the response letter, one regression per transfer level measured in the strategy method.

3.2. It is not very intuitive to focus on the intercept for the effects of interest. Perhaps start with a non-parametric test for the equality of proportions in choosing the masked or unmasked avatar to allocate the largest amount. This may give the reader some intuition on why you focus on the intercept in the main regression analysis from Experiment 3.

4. Minor comments

4.1. Figures appear with low resolution. Please fix this.

4.2. PLOS ONE requires data availability, but this is not currently satisfied in the submission. Please fix this.

References

Johnsen, Å. A., & Kvaløy, O. (2016). Does strategic kindness crowd out prosocial behavior? *Journal of Economic Behavior & Organization*, *132*, 1-11.
