# Supplementary material for: How facemasks shape trust in social interactions
Source: PLoS One. 2025 Sep 12;20(9):e0331918. doi: 10.1371/journal.pone.0331918 (PMC12431196; doi:10.1371/journal.pone.0331918)
Supplement: Attachment — Submitted filename: Response to Reviewers.docx [file pone.0331918.s012.docx]

Reply to reviewer comments:

1. Concerns on the use of faces from a databank and hypothetical games.

My main concern with the overall manuscript is an alternative explanation for why Experiments 1 and 2 yielded null results: the combination of hypothetical choices with preexisting faces where the facemask was superimposed may have created an artificial decision environment, and respondents did not take it seriously.

1.1. Did you ask prolific respondents if they were familiar with these faces? As they are well-trained participants for this type of study, if they perceive the task as being too artificial, you may have more noisy responses that ultimately decrease your ability to detect effects. Please clarify if this question was asked, and discuss this in the paper.

We appreciate this comment, since it points to something we had not considered, but, as we explain, we think there is little threat of participants recognising any of the faces.

Firstly, there are more than 600 faces in the Chicago Face Database (CFD). This includes a main set of 597 neutral faces and an additional 88 multiracial faces, and 142 Indian faces. There are, in addition, multiple images from many faces showing a range of emotions. We used only 8 images in total. Even if some Prolific participants had previously taken part in studies using images from the CFD, the likelihood that they saw any of these images would be small. We have revised the manuscript to include the number of faces contained in the CFD, and added the following text (p. 9):

“Because we did not want visible facial features and expressions to confound the influence of facemasks, we selected four male and four female faces using a procedure that minimised differences in age, attractiveness and trustworthiness as recorded in the CFD. These faces were chosen to look like ordinary people participants would routinely see in their daily lives (their CFD unusualness ratings ranged from 2.1 to 3.1 on a 1-7 scale, 1 = Not at all; 7 = Extremely). Moreover, because of the large size of the CFD, participants would most likely be unfamiliar with any of the specific faces we chose.”

This text is followed by the following footnote, which describes a search we conducted using Google Scholar:

“We found 73 studies mentioned on Google Scholar that used items from the CFD on Prolific. Of these, 32 were conducted on UK samples. These typically involved small samples of participants (100 or so) and a small number of faces. Given that Prolific has around 80,000 unique and active UK participants, the likelihood that our participants would recognise any of these faces is negligible.”

The reviewer is also concerned about the artificiality of the decision context, especially in relation to the mask manipulation. Superimposing a mask on a preexisting photograph is a method for obtaining strict stimulus control in facial perception research (e.g., same individual features, lighting, and angle; only the presence/absence of the mask differs – see, e.g., Hies & Lewis, 2022; Bassiri-Tehrani et al., 2022; Light et al., 2024). We now mention how this is a commonly used method and cite this literature on p. 9.

References:

Hies, O., & Lewis, M. B. (2022). Beyond the beauty of occlusion: Medical masks increase facial attractiveness more than other face coverings. Cognitive Research: Principles and Implications, 7, 1-6.

Bassiri-Tehrani, B., Nguyen, A., Choudhary, A., Guart, J., Di Chiaro, B., & Purnell, C. A. (2022, January). The effect of wearing a mask on facial attractiveness. In Aesthetic Surgery Journal Open Forum (Vol. 4, p. ojac070). US: Oxford University Press.

Light, A. E., Reidinger, B., Henderson, M. A., & Ma, D. S. (2024). Differential impacts of face masks for threat evaluations of Asian versus Black faces. Group Processes & Intergroup Relations, 27(4), 759-778.

1.2. I also believe that this issue of how artificial the decision looked may partly explain why those who decided first as the trustors were less prosocial: starting with the second decision reinforced the hypothetical nature of this trust game, and therefore, they could feel that their choices were less consequential toward others.

Although the hypothetical nature of the task was known from the outset, it is possible that facing a second decision may have reinforced this understanding. However, the likely effect of this would be that people would become less selfish, because they realise there is no cost to this generosity. Our results go in the opposite direction with participants being more selfish in the second round. This is in line with prior research showing that players who are aware from the start that they will be playing both roles in the trust game often become more self-interested (Burks, Carpenter & Verhoogen, 2003). To control for this potential bias, we did not tell participants that they would be playing both roles until after they made their first decision. We have now clarified this on p. 7, where the following text is found:

“Evidence suggests that people who learn in advance they will be playing both roles in the trust game typically become more selfish [35], so we minimised this order effect by not announcing the presence of a second decision until it was encountered. ”

Importantly, our observed order effects do not interact with the mask manipulation, so they do not affect the interpretation of our main tests. Moreover, because we withheld the information about the second decision while the first was being made, we can treat the first decision as if it came from a between-subjects experiment. If we conduct our analysis only on the first decisions, we find the same results: masks do not affect the amount sent by trustors. The same holds for an analysis restricted to the second decisions. We have added these analyses to the Supplementary Materials. We summarise them briefly on p. 10-11 as follows:

“In both experiments, the amount sent by trustors was lower if they first took the role of trustee (Exp 1: t(710) = 4.61, 95% CI [0.61, 1.52], d = 0.33, p < .001; Exp 2: t(356) = 4.57, 95% CI [0.88, 2.20], d = 0.47, p < .001). Separate analyses conducted on the first and second decisions similarly show no effect of the mask manipulation (see Supplementary Materials).”

Reference:

Burks, S. V., Carpenter, J. P., & Verhoogen, E. (2003). Playing both roles in the trust game. Journal of Economic Behavior & Organization, 51(2), 195-216.

1.3. The use of the strategy method in the trust game could also explain the behaviour of the second mover, reinforcing why those starting with this choice behaved differently. See Johnsen and Kvaløy (2016).

We thank the reviewer for pointing out the similarity of our Experiment 1 result and Johnsen and Kvaløy’s findings. We now mention that similarity explicitly, and, as for 1.2 above, conduct further analyses to show that the effect does not interact with our mask manipulation. These changes are reflected by our revised text on p. 13:

“In Experiment 1, participants who played as trustee first returned on average 2.5% more than those who played as trustor first. This effect – akin to Johnsen and Kvaløy’s [43] finding of less reciprocation of trust if it was announced to participants that they would play two repetitions of the game than if it was not – did not interact with our mask manipulation (see Supplementary Material).”

Reference:

Johnsen, Å. A., & Kvaløy, O. (2016). Does strategic kindness crowd out prosocial behavior?. Journal of Economic Behavior & Organization, 132, 1-11.

1.4. I also recommend that the authors justify their choice of a hypothetical trust game based on existing literature following the same incentive decision.

Because we wanted to have full control over the appearance of the counterparts in the trust game (which we achieved by using images from the CFD), we could not incentivise behaviour without deceiving our participants. We hence conducted the game hypothetically. However, evidence shows that behaviour in the trust game does not differ between comparable incentivised and hypothetical versions (e.g., Thielmann et al., 2016). We have added this clarification and a reference to this work on p. 6 and fn. 1.

“As detailed in the next section, our manipulation of the counterpart consisted in showing pictures of a hypothetical coplayer. For this reason, we could not provide truthful real-time feedback on the outcome of the game, nor could we link the participants’ decisions in the game to monetary incentives.”

Footnote: “Thielmann et al. [33] show that behaviour in hypothetical trust games is indistinguishable from behaviour in real-incentive versions when all other design features are kept constant.”

Reference:

Thielmann, I., Heck, D. W., & Hilbig, B. E. (2016). Anonymity and incentives: An investigation of techniques to reduce socially desirable responding in the Trust Game. Judgment and Decision making, 11(5), 527-536.

2. Design choices to explain better.

2.1. The design in Experiment 3 is interesting, but it must be better explained. Although the use of two simultaneous second movers is clever, the fact that the transferred amount does not increase must be better explained. At first sight, this game variation does not measure trust but instead generosity because there is no reason to give money back if it is purely redistributive. The key issue with the game is that the sender has no money left, so she *trusts* that the recipient of the largest amount would send some money back. This must be made clearer in the text.

We thank the reviewer for giving us the opportunity to clarify this. We chose this method because we found it simple to explain to participants. Your intuition is exactly ours. To make this clearer, we now have the following text on p. 18.

“A further distinction between Experiment 3 and the earlier ones is in the trusting decision by the first mover. To simplify the task, we did not multiply the amount sent but left it unchanged, so that trustworthiness concerned how much of this money would be returned. The difference between participants is that one would have received £20, and the other £10. Because the trustor could not keep any money for themself, their decision concerning who would receive the £20 was a decision about who could be trusted to send back more of the money. In this case, either the masked or unmasked person.”

2.2. In Experiments 1 and 2, it is not clear why participants played the trust game a single time. This limited the authors’ possibilities of computing some helpful within-subjects measurements. Learning how to play the trust game was a high cost for respondents, so that they could then apply it more than once. I would like to hear the authors’ thoughts on this.

In most experiments that involve repetitions of the same game, participants are either provided with feedback about the previous game between repetitions, or the parameters of the game change between repetitions. In our case, given the absence of a real counterpart, providing truthful feedback between repetitions would have been impossible. The nature of the trust game is such that, in relation to our research questions, there isn’t much to be learnt from altering the parameters of the game. Applying the mask manipulation within-subjects in the context of a hypothetical task like ours could have generated experimenter demand effects. Our third experiment made direct comparisons between masked and unmasked counterparts possible in a way that is much less likely to generate such confounding effects. In the revised version of the manuscript, we clarify why the game was not repeated when we introduce it on p. 6:

“The game was played only once. The characteristics of the counterpart (i.e., masked and unmasked) and the gender of the participant were our main independent variables. As detailed in the next section, our manipulation of the counterpart consisted in showing pictures of a hypothetical co-player. For this reason, we could not provide truthful real-time feedback on the outcome of the game, nor could we link the participants’ decisions in the game to monetary incentives. For the same reason, the game was played only once, as is common in the literature [9, 31, 32].”

2.3. Why—in Experiment 2—the sex restriction kept female counterparts instead of male counterparts? Please explain.

In Experiment 1, we found that the gender of the counterpart had no effect on the behaviour of either trustors or trustees, so to use our research budget more efficiently, we focused only on one of the genders. Because behaviour did not differ, it did not matter which one we decided to keep. We have revised the wording of our manuscript to reflect this (p. 8):

“Second, since the gender of the hypothetical counterpart had no influence on the behaviour of either trustors or trustees in Experiment 1, we restricted Experiment 2 to female counterparts only. That the gender of the trustee has no effect on trust and trustworthiness is consistent with a recent meta-analysis [37].”

2.4. In Experiment 2, the 10-second exposure raises salience of the mask, but it may have also raised the experimenter demand effect. A discussion on this would help the reader clarify this design feature.

Demand effects are always a possibility in experimental work. In this case, however, it can be argued that, in relation to our main research questions, they may work either way. It is possible that people saw the masked face and thought they should give more or return more. But it is also possible that the mask may cause a reactance effect, reducing the amount sent or returned. However, because we did not obtain a difference between conditions, such effects are unlikely to have played a role. As we now clarify on p. 8, this design decision was meant to ensure that participants engaged with the picture of the hypothetical counterpart:

“… to increase the salience of the counterpart’s face, we required participants to view their picture for at least 10 seconds at the start of the experiment.”

3. Statistical analysis

3.1. Is the effect of the facemask null for every transfer level? I suggest reporting, in the

appendix or at least in the response letter, one regression per transfer level

measured in the strategy method.

Thank you for this suggestion. We ran separate regressions, which we now report in the supplementary materials. These additional analyses confirm that, when controlling for participant gender, counterpart gender and order of play, the null result is very consistent across transfer levels. We summarise this in p.13 fn. 3:

“As a robustness check, Table S12 in the Supplementary Material reports separate regression estimates for each amount that could be sent by the trustor. When controlling for participant gender, counterpart gender, and order of play, the proportion returned by the trustee is consistently unaffected by the mask manipulation.”

3.2. It is not very intuitive to focus on the intercept for the effects of interest. Perhaps start with a non-parametric test for the equality of proportions in choosing the masked or unmasked avatar to allocate the largest amount. This may give the reader some intuition on why you focus on the intercept in the main regression analysis from Experiment 3.

We acknowledge that it is uncommon for the intercept in a regression to be the most important variable of interest, but it is the appropriate analysis in this case. In line with the principles of open science, we decided to stick with our pre-registered test. We have amended our text to explain why we are primarily interested in the intercept (p. 20):

“We ask whether the proportion allocating £20 to the masked counterpart exceeds 50%, and whether this depends on the participant’s gender and the task frame. As pre-registered, we do this using logistic regressions (see Table 3) where the dependent variable is whether the masked counterpart was allocated £20. Because our benchmark is 50%, the main variable of interest is the intercept.”

Given how high the proportions of participants trusting the masked counterpart are, unsurprisingly, non-parametric tests confirm the results of our regression by strongly rejecting the null hypothesis that the proportion is equal to 50% (e.g., p < 0.001 in an exact binomial test or in a Chi-squared test).
